# Supplementary material for: Pregnancy with mixed connective tissue disease: Exploration of factors influencing live birth outcomes
Source: PLoS One. 2024 Dec 5;19(12):e0303318. doi: 10.1371/journal.pone.0303318 (PMC11620383; doi:10.1371/journal.pone.0303318)
Supplement: Attachment — Submitted filename: Response to Reviewers.docx [file pone.0303318.s002.docx]

Re: Manuscript ID: PONE-D-24-14916

September 16, 2024

Emily Chenette

Editor in Chief, *PLoS One*

Thank you for giving us the opportunity to submit a revised draft of our manuscript titled, “**Pregnancy with mixed connective tissue disease: Exploration of factors influencing live birth outcomes”** to *PLoS One*.

We appreciate the time and effort that you and the reviewers have dedicated to providing your valuable feedback on our manuscript. We are grateful for their insightful comments. We have been able to incorporate changes to reflect most of the suggestions provided by the reviewers, and we believe these changes have greatly improved the quality of our manuscript. We hope that with these changes, the manuscript meets with your and the reviewers’ approval.

Please find attached herewith our point-by-point responses to the reviewers’ comments. Revisions in the manuscript have been indicated with yellow highlighted text. The page and line numbers have been transcribed in accordance with the tracked version of the manuscript.

Many parts of the manuscript have been carefully rewritten to enhance the flow and clarity of some previously ambiguous text. We hope that these revisions and our accompanying responses would bring our manuscript closer to publication in the *PLOS ONE.*

**Point-by-point response to the reviewers' comments**

We would like to thank the Reviewer for spending your valuable time to review our manuscript and the constructive critique to improve our manuscript. The manuscript has benefited immensely from the suggestions provided. We have made every effort to address the issues raised and to respond to all the comments and suggestions and have made the necessary changes in accordance with the suggestions and believe that the changes made in this round of revision would satisfactorily address the concerns raised. Please find our response to each item below.

*1) The combined case-series and case-control design allows for a detailed examination of both individual cases and broader patterns, which is a commendable approach. The rationale for choosing this design is not explicitly stated. Clarifying why this design was preferred over others would strengthen the methodological justification.*

**Response：** Thank you for your insightful comment. We chose this study design considering the low prevalence of MCTD-complicated pregnancies. The case series design allows for the detailed collection and analysis of individual cases, whereas the case-control study is well-suited for examining exposures and identifying broader patterns and trends. By comprehensively evaluating both distinct cases and more generalizable results, we aim to deepen the understanding of MCTD-complicated pregnancies. We have added this explanation to the manuscript in method part.

**Line 61-64：**”This study design was selected considering the low prevalence of MCTD-complicated pregnancies. The case series design allows for the detailed collection and analysis of individual cases, whereas the case-control study is well-suited for examining exposures and identifying more generalizable results.”

*2) No information is provided on how data consistency was maintained over this long period. The potential for changes in diagnostic criteria, treatment protocols, or data recording practices over time should be addressed.*

**Response:** Thank you for highlighting the importance of data consistency over the extended study period. We took several measures to ensure data consistency, including regular data audits, standardization of diagnostic criteria based on the guidelines available at the time of data collection, and consistent alignment of treatment protocols with these guidelines. We have now added these details to the revised manuscript.

**Line 98-101：**“To ensure data consistency during the extended study period, we conducted regular data audits. Specifically, we confirmed that diagnostic criteria were standardized based on the guidelines available at the time of data collection, and treatment protocols were consistently aligned with these guidelines.”

*3) The exclusion of non-Asian women limits the generalizability of the findings. The justification for this exclusion should be elaborated. Additionally, the handling of suspected MCTD cases should be clarified—what criteria were used to exclude them, and how were borderline cases managed? The live-birth and non-live birth groups are well-defined, but further explanation on how miscarriages and stillbirths were confirmed and categorized would enhance clarity.*

Response:

Thank you for your valuable feedback. The exclusion of non-Asian women was based on the study’s objective to investigate a specific population where cultural and genetic factors, such as immune cell gene expression[9], might influence the outcomes.

The exclusion criteria for suspected MCTD cases were based on the diagnostic criteria published in 1987 by Kasukawa et al., as revised in 1996 and 2004 by the representatives of the MCTD Research Committee of the Japanese Ministry of Health and Welfare. Additionally, all cases were reviewed by rheumatology specialists to ensure there were no discrepancies in the diagnosis and treatment of MCTD.

Miscarriages and stillbirths were classified based on gestational age, and all cases were confirmed through routine prenatal examinations. These classifications were verified through medical records, and imaging findings used in diagnosis were also reviewed to maintain data accuracy. We have added these clarifications to the manuscript.

**Line 67-70:** “Miscarriages and stillbirths were classified based on gestational age, and all cases were confirmed via routine prenatal examinations. These classifications were verified through medical records. Additionally, imaging findings used in diagnosis were reviewed to maintain data accuracy.”

**Line 79-82:** “The exclusion of non-Asian women was based on the study’s objective to investigate a specific cohort where cultural and genetic factors, such as immune cell gene expression[9], might influence the outcomes.”

Reference: 9, Yazar S, Alquicira-Hernandez J, Wing K, Senabouth A, Gordon MG, Andersen S, et al. Single-cell eQTL mapping identifies cell type-specific genetic control of autoimmune disease. Science. 2022;376: eabf3041. doi:10.1126/science.abf3041

*4) Describe the data collection process in more detail. How were patient records accessed and verified? How was data consistency ensured over the extended study period?*

Response: Thank you for your comments regarding the data collection process. We retrieved patient records from a secure and comprehensive database system, with all data accessed and confirmed after receiving approval from the relevant ethics committee. The data used in the study were double-checked by multiple obstetricians and rheumatologists to ensure accuracy.

To maintain data consistency over the long study period, regular audits and data cleaning were conducted, and diagnostic criteria and treatment protocols were standardized according to the latest guidelines. (as also noted in our response to question 2).

We have now included these details in the manuscript.

**Line 95-97:** “Patient information were retrieved from a secure and comprehensive database system, with approval from the Ethics Committee. To ensure accuracy, multiple obstetricians and rheumatologists verified all the data utilized in the study. “

**Line 98-101：**“To ensure data consistency during the extended study period, we conducted regular data audits. Specifically, we confirmed that diagnostic criteria were standardized based on the guidelines available at the time of data collection, and treatment protocols were consistently aligned with these guidelines.” **(as also noted in our response to question 2).**

*5) In the result section, there is no sufficient information on immunology (autoantibodies, including APL, anti-DNAds, anti-SM, anti-SSA/Anti-SSB), type of immunossupression before pregnancy, doses of corticosteroids before and during pregnancy and the clinical manfiestations of the MCTD, including PH, which is referenced as being one of the causes for pregnancy morbidity.*

Response: Thank you for your feedback. We have added more detailed information on autoantibodies, immunosuppressive treatment, corticosteroid dosage before and during pregnancy, and the clinical manifestations of MCTD. Specifically, all MCTD cases were confirmed to be U1-RNP positive. Among the SLE-complicated cases, 8 were positive for ds-DNA antibodies, though none had anti-Sm antibodies or severe organ damage such as lupus nephritis, and no cases of kidney failure were observed. Additionally, one case was positive for cardiolipin antibodies, but no cases met the criteria for APS-related syndromes. Eight cases were positive for anti-SSA antibodies, but none of the newborns developed congenital heart block. Regarding immunosuppressive treatment, four cases involved the use of tacrolimus. There were no adjustments in prednisolone dosage during pregnancy, but two cases had increased doses postpartum. The clinical manifestations of MCTD included pulmonary hypertension in two cases, with no reports of kidney dysfunction. We have incorporated these details into the manuscript.

**Line 107-118:** “Regarding MCTD management during pregnancy, 29 cases (50.9%) used prednisolone, and 4 (7.0%) used immunosuppressants (Table 1). In all four cases, tacrolimus was used. As for complications, two cases developed PH, with no cases of kidney dysfunction (Table 1). Eight cases also developed complications in SLE, although all were negative for anti-Smith antibodies and showed no severe organ involvement like lupus nephritis (Table 1). Although these cases fulfilled the American College of Rheumatology criteria for SLE, MCTD was diagnosed based on their clinical manifestations, such as scleroderma symptoms and myositis. All cases were confirmed to be U1-RNP positive. Additionally, one case was positive for cardiolipin antibodies, but no cases met the criteria for antiphospholipid-related syndromes. Eight cases were positive for anti SS-A antibodies, but none of the newborns developed congenital heart block (Table 1).”

*6) "However, the live birth group exhibited a significantly higher rate of prednisolone use (62.2% vs. 30.0%, p=0.02), while the non-live birth group showed a greater prevalence for higher doses of prednisolone (median dose: 7 vs. 10 mg/day, p=0.03) (Table 2)". This sentence is very confusing and the data the authors present is insuficient to get this conclusion. Does this mean that corticosteroid use is associated with favorable outcomes? But doses higher than 10 mg/day were associated with unfavourable outcomes?*

Response: Thank you for pointing out the potential confusion in the original phrasing. We have revised the text in the Results section to clarify our findings. Specifically, we now state that “the live birth group exhibited a significantly higher rate of prednisolone use (62.2% vs. 30.0%, p=0.02). **In contrast, when focusing on the patients who used steroids,** the non-live birth group had a statistically significantly higher median dose of prednisolone (7 vs. 10 mg/day, p=0.03).” This revision should clarify that while prednisolone use was more common in the live birth group, higher doses were more prevalent in the non-live birth group. We have also discussed this in the Discussion section.

**Line 131-136:** “However, the live birth group exhibited a significantly higher rate of prednisolone use (62.2 vs. 30.0%, p=0.02). In contrast, when dosage of steroids was compared among the patients using steroids, the non-live birth group had a statistically significantly higher median dose of prednisolone compared to that of the live birth group (10 vs. 7 mg/day, p=0.03). (Table 2).”

*7) "Conversely, no significant differences were observed in other immunosuppressant, low-dose aspirin, and heparin uses between the two groups (Table 2)." There is no mention of the type of immunosuppressive treatment used and this is important.*

Response：Thank you for your valuable feedback. As mentioned above, the only immunosuppressants used by patients during the study period was tacrolimus. The details have been provided in the earlier section. Additionally, as shown in Table 2, there was no statistically significant difference in the usage rates between the two groups.

We have added the following sentence: Line 111-112: “In all four cases, tacrolimus was used.”

*8) The discussion lacks depth in comparing the findings with a broader range of studies. It relies heavily on a few sources, which may limit the scope of the comparison.*

Response: Thank you for your valuable feedback. We appreciate your suggestion to broaden the scope of our discussion by comparing our findings with a wider range of studies. In response, we have added additional references, including both case series and systematic literature reviews, to provide a more comprehensive comparison of our results.

Specifically, we have included references that highlight the high incidence of fetal complications in pregnancies complicated by MCTD, such as prematurity (48.1%) and intrauterine growth restriction (38.3%). This information has been integrated into the discussion to provide a broader context for our findings and to strengthen the overall comparison with other studies.

Additionally, we would like to express our gratitude for the comments provided in points No.9 and 10. These suggestions have allowed us to further refine our discussion, making it more nuanced and comprehensive.

We believe that these additions enhance the depth of our discussion and provide a more nuanced understanding of the challenges associated with MCTD-complicated pregnancies.

Thank you again for your thoughtful comments, which have contributed to improving the quality of our manuscript.

**Line 227-235:** “Regarding specific delivery outcomes, our study reported a significantly higher preterm birth rate compared to the national average of 5.0% and other Asian rates [22, 23]. Similarly, previous studies have reported high rates of fetal complications in MCTD-complicated pregnancies, with prematurity observed in 48.1% and intrauterine growth restriction in 38.3% of cases [24]. Although the reasons for increased FGR incidence in MCTD-complicated pregnancies remain unclear, a mechanism similar to the increased incidence of FGR/SGA (approximately 10–30%) in SLE may be possible. Particularly, the increased occurrence of elevated blood pressure and increased blood [25, 26].”

Reference：

24, Tardif M-L, Mahone M. Mixed connective tissue disease in pregnancy: A case series and systematic literature review. Obstet Med. 2019;12: 31–37.

*9) There is no mention of these important guidelines:*

*Götestam Skorpen C, Hoeltzenbein M, Tincani A, et alThe EULAR points to consider for use of antirheumatic drugs before pregnancy, and during pregnancy and lactationAnnals of the Rheumatic Diseases 2016;75:795-810.*

*Andreoli L, Bertsias GK, Agmon-Levin N, et alEULAR recommendations for women's health and the management of family planning, assisted reproduction, pregnancy and menopause in patients with systemic lupus erythematosus and/or antiphospholipid syndromeAnnals of the Rheumatic Diseases 2017;76:476-485.*

Response：Thank you for your valuable feedback. We have revised the Discussion section to incorporate guidelines from The European Alliance of Associations for Rheumatology (EULAR) Points to Consider for Use of Antirheumatic Drugs Before Pregnancy, and During Pregnancy and Lactation [17] as well as the EULAR Recommendations for Women’s Health and the Management of Family Planning, Assisted Reproduction, Pregnancy, and Menopause in Patients with Systemic Lupus Erythematosus (SLE) and/or Antiphospholipid Syndrome (APS) [19]. Specifically, we have referenced these guidelines to support the use of antirheumatic drugs like prednisolone during pregnancy, emphasizing the importance of continuing certain corticosteroids to effectively manage disease activity while minimizing risks to both the mother and fetus [17]. Additionally, we aligned our findings with the EULAR recommendations, which advise minimizing the use of high-dose steroids during pregnancy to reduce the risk of adverse outcomes [19]. The guidelines further emphasize the importance of combination therapy with other immunosuppressive agents to achieve optimal disease control while minimizing steroid dosage [19]. This addition strengthens the discussion by aligning our findings with established international recommendations.

**Line 206-211:**” Similarly, prednisone has been observed to effectively manage disease activity and contribute to better delivery outcomes in other CTDs, such as SLE [16]. This is consistent with the European Alliance of Associations for Rheumatology (EULAR) points to consider for the use of antirheumatic drugs during pregnancy, which recommend continuation of certain corticosteroids like prednisolone for effective management of disease activity [17].

**Line 216-226:** “Our results were consistent with those of previous studies, showing that doses >10 mg/day resulted in unfavorable live birth outcomes. In line with the EULAR recommendations, our findings support minimizing the use of high-dose steroids during pregnancy to reduce the risk of adverse outcomes [19]. The guidelines, along with our study, emphasize the importance of combination therapy with other immunosuppressive agents to achieve optimal disease control while minimizing steroid dosage [19]. Studies have also reported that high-dose steroids can increase the risk of placenta accreta formation and uterine myometrial fibrosis due to reduced estrogenic effect and endometrial thinning, potentially causing atonic bleeding and substantial blood loss [19, 20]. Thus, minimizing the total steroid dose whenever possible is crucial to ensure optimal outcomes for both the mother and fetus.”

Reference

17, Götestam Skorpen C, Hoeltzenbein M, Tincani A, Fischer-Betz R, Elefant E, Chambers C, et al. The EULAR points to consider for use of antirheumatic drugs before pregnancy, and during pregnancy and lactation. Ann Rheum Dis. 2016;75: 795–810.

19, Andreoli L, Bertsias GK, Agmon-Levin N, Brown S, Cervera R, Costedoat-Chalumeau N, et al. EULAR recommendations for women’s health and the management of family planning, assisted reproduction, pregnancy and menopause in patients with systemic lupus erythematosus and/or antiphospholipid syndrome. Ann Rheum Dis. 2017;76: 476–485.

-------------------

*10) The discussion provides a detailed analysis of the impact of steroid use on pregnancy outcomes, highlighting both positive effects (improved live birth rates) and negative effects (higher doses associated with unfavorable outcomes). The explanation of the mechanisms by which steroids might improve outcomes (e.g., suppression of autoantibodies, downregulation of natural killer cells) is somewhat speculative and could benefit from more robust evidence or references.*

Response: Thank you for your insightful comments on our manuscript. We appreciate your feedback regarding the speculative nature of the explanation involving the suppression of autoantibodies and its impact on pregnancy outcomes. Based on your suggestion, we have revised the discussion to remove the section on the suppression of autoantibodies, which was indeed speculative.

Instead, we have incorporated findings from recent studies, particularly referencing Robertson et al. (2016), to provide a more robust and evidence-based explanation of how prednisolone may influence pregnancy outcomes in women with MCTD [14]. The revised text now discusses the potential for prednisolone to shift the immune response balance towards M2 macrophages, tolerogenic dendritic cells, and a stronger regulatory T cell response, particularly in women predisposed to excessive inflammation and immunity, such as those with MCTD [14].This mechanism is proposed as a contributing factor to the lower stillbirth rates observed in our study among steroid users.

We believe this revision strengthens the discussion by grounding our hypotheses in more recent and relevant literature, and we appreciate your guidance in improving the quality of our manuscript.

**Line 192-208:** ”Although relatively higher MCTD activity was expected in steroid users, our study observed lower stillbirth rates in patients who used steroids. Recent studies suggest that the effects of prednisolone on the immune system may vary depending on the individual’s immune status [14]. As highlighted in previous reviews, although suppression of peri-conception inflammation might impair immune function in some women, it is possible that in women predisposed to excessive inflammation and immunity, such as those with MCTD, prednisolone could help shift the balance of the immune response towards M2 macrophages, tolerogenic dendritic cells, and a stronger regulatory T cell response, thereby promoting a more favorable pregnancy outcome [14]. Steroid use in patients with unexplained recurrent pregnancy loss has been reported to downregulate natural killer cells in the uterine endometrium, thereby reducing miscarriage rates [15]. It is therefore possible that a similar mechanism contributed to the prevention of stillbirth with steroid use in MCTD. This aligns with previous studies that has shown that a combination of azathioprine and steroids significantly improves pregnancy outcomes in MCTD [5]. Similarly, prednisone has been observed to effectively manage disease activity and contribute to better delivery outcomes in other CTDs, such as SLE [16].”

Reference:

14, Robertson SA, Jin M, Yu D, Moldenhauer LM, Davies MJ, Hull ML, et al. Corticosteroid therapy in assisted reproduction - immune suppression is a faulty premise. Hum Reprod. 2016;31: 2164–2173.

*11) The limitations could be expanded to discuss more potential biases and confounding factors. The reliance on clinical judgment for steroid use and the retrospective, single-center design are mentioned, but their implications on the results could be explored further.*

Response：Thank you for your insightful comments. We agree that the limitations section could be expanded to discuss potential biases and confounding factors in more detail. Specifically, the reliance on clinical judgment for steroid use introduces a potential bias, as decisions may vary based on the experience and knowledge of the attending physician. This variability could have influenced the dosage and administration of steroids, thereby affecting pregnancy outcomes.

Additionally, the retrospective, single-center design of the study poses limitations such as selection bias and information bias. Since data were collected from past medical records, there may be inconsistencies in data recording and variations in clinical practices over time. These factors could have influenced the results and should be considered when interpreting the findings.

We have revised the limitations section to include these considerations, acknowledging that these biases may have impacted the study outcomes and suggesting ways to address these issues in future research.

**Line 245-257:** "Despite the insights offered in this study, certain limitations should be acknowledged. First, there was limited generalizability of findings due to the study’s retrospective, single-center design. This design introduces limitations such as selection bias and information bias, as data were collected from past medical records. The study results could have been influenced by changes in clinical practices and variations in data recording over time. Second, steroid use solely relied on clinical judgment, resulting in the lack of a standardized steroid administration protocol. This reliance on clinical judgment introduces potential bias, as the decisions regarding dosage and administration may vary depending on the physician’s experience and knowledge, which could have influenced the pregnancy outcomes observed in this study. Third, the study only focused on the maximum dosage of steroids. Future studies might benefit from including the cumulative dosage to further elucidate the association between steroid use and MCTD-complicated pregnancy."

*12) The discussion on future research is brief. It should include more detailed recommendations for how future studies can build on these findings, including suggestions for multicenter studies or standardized protocols for steroid use.*

Response: Thank you for your insightful comment. We agree that the discussion on future research could be expanded to provide more detailed recommendations. We have revised the text to include suggestions for multicenter studies, which could enhance the generalizability of the findings by incorporating a more diverse patient population. Additionally, we recommend the development and implementation of standardized protocols for steroid use in MCTD-complicated pregnancies. Such protocols could help to minimize variability in treatment and provide more consistent data on the effects of steroid use. Furthermore, future studies should consider including cumulative steroid dosage to further elucidate its association with pregnancy outcomes in MCTD-complicated pregnancies.

**Line 255-262:** “Future studies might benefit from including the cumulative dosage to further elucidate the association between steroid use and MCTD-complicated pregnancy. Additionally, multicenter studies are recommended to enhance the generalizability of the findings by incorporating a more diverse patient population. The development and implementation of standardized protocols for steroid use in MCTD-complicated pregnancies are also crucial. Such protocols would help to minimize variability in treatment and provide more consistent data on the effects of steroid use.”

In addition to the above comments, all spelling and grammatical errors have been corrected.

Thank you once again for your valuable comments and suggestions. We hope you find these revisions are up to your expectations.

Sincerely,

Tsukasa Yoshida, Jun Takeda, Sumire Ishii, Masakazu Matsushita, Naoto Tamura and Atsuo Itakura

Corresponding author:

Jun Takeda, Tsukasa Yoshida

Department of Obstetrics and Gynecology

Juntendo University Faculty of Medicine

2-1-1 Hongo, Bunkyo-ku

Tokyo, 113-8421, Japan

Email: t.yodhifs.wa@juntendo.ac.jp

Tel: 03-3813-3111
